# Meeting in Leeds: Local Centre to Be Formed

**Published:** 1918-05-04

**Authors:** 


### THE COLLEGE OF NURSING.

### Meeting in Leeds: Local Centre to be Formed.

At a largely attended meeting of nurses held in Leeds on Monday, April 29, it was decided to form a local centre in connection with the College of Nursing. The meeting, which was open to "all interested in the nursing profession," was presided over by Alderman Charles Lupton, Chairman of the Leeds General Infirmary. It was hoped that the Hon. Sir Arthur Stanley, M.P., Chairman of the College, would be present, but he sent word that he had to fulfil an important engagement in Switzerland, and was sorry he could not attend. In his stead Miss Cox-Davies, R.R.C., Matron of the Royal Free Hospital, and a member of the Council of the College, explained the chief principles animating those behind the movement; and the meeting was also addressed by Miss Cowlin, the newly appointed Organising Secretary of the College. Others present included the Lady Mayoress of Leeds (Mrs. Frank Gott), Major-General Sir Berkeley Moynihan, C.B., Miss Sparshott (Royal Infirmary, Manchester), Miss Innes (General Infirmary, Leeds), Miss Spann (Leeds Township Infirmary), Dr. Hellier, Dr. Eddison, Mr. Grosvenor Talbot, Mr. F. G. Bray (manager of the Leeds Infirmary), and some 200 nurses.

The Chairman, **Mr. CHARLES LUPTON,** remarked that the meeting had been called to put into practical effect a resolution passed at a large and representative meeting in Leeds some few months ago favouring the establishment of a College of Nursing. The question now was whether a local centre should be set up in Leeds, as to which he supposed there could not be much difference of opinion. Many criticisms had been levelled at the foundation of the College, but none of those criticisms had convinced him that the policy of the founders was wrong. At the same time, care should be taken on one point. They should not endeavour to subject every nurse to absolutely the same type of training, and, furthermore, they ought not to make the mission of all nurses dependent entirely upon an examination on that training. The spheres of nursing—some of which he described—were many and varied, and each of them required special vocational training, but not the same training. He, therefore, urged the Council to consider whether these requirements could not be met first of all by general training in a well-equipped hospital, after which nurses could go forward to some specialised training and be labelled upon the results of that advanced training. To ensure this he thought the provinces should be heard in the counsels of the College; they should have strong representation proportionate to their numbers.

**Miss COX-DAVIES** urged that it was the duty of the nurses themselves to direct and shape the policy of the College. "Nobody," she said, "can do it for you. The College has been brought into existence, and has already got very good substantial roots. It now lies with you, the nurses of the United Kingdom, to make it your own and to make it worthy of the profession to which you belong." The Register now contained the names of 8,000 nurses, and nomination papers for the Council had gone out. It would be extremely interesting to know how many of those papers had been filled up. Now was the time to determine the constitution of the Council, and if in two or three years' time there was grumbling it would be their own fault and the fault of the representatives about to be elected. Such a chance as this had never been within the grasp of the nursing profession before. "Don't let us throw it away."

Discussing the vital aims of the College, Miss Cox-Davies mentioned first the organisation of the nursing profession, as to which there was no difference of opinion; and, secondly, the securing of a Bill for State Registration, as to which, she said, the profession must arrive at some form of general agreement. So long as they wrangled and quarrelled there was absolutely no chance of getting the Bill. For its part the College of Nursing had never ceased its efforts at agreement, and they yet hoped that some real, substantial ground of agreement would be reached. The third of the vital aims was the one-portal examination, with which, she gathered, the Chairman was somewhat at variance, though there could be no objection even here to the basic principle that a certain minimum of knowledge and experience should be secured as a protection for the public. Fourthly, came a standard training and a uniform curriculum, extending over a minimum of three years, and comprising a definite amount of medical and surgical work. Miss Cox-Davies spoke of the rapid advance in the size of the Register during the past year—an advance from 2,000 to 8,000, and expressed the hope that the end of another year would bring the number up to 18,000. She warmly commended the establishment of a local centre in Leeds, with its club-rooms and educational equipment, providing possibly for post-graduate courses—a place where nurses would get away from the monotony of their everyday surroundings, and from the cloy of "shop" talk. Important, too, was the question of finance, and in this connection she mentioned the work of the British Women's Committee and the progress of the "Tribute" Fund, denying that help of this kind carried in its acceptance by nurses any taint of "charity," as had been said. It had also been said that the College Register was not going to be much good to the profession, because women were being admitted to it who did not hold certificates of three years' training—women, in fact, who had only done war work. She took this opportunity of saying that the statement thus outlined was absolutely and entirely untrue. Of the 8,000 women admitted there was not one who had not complied with the regulations laid down by the College, and she had a right to say this because she had been Chairman of the Registration Committee practically ever since the College was formed.

**Major-General SIR BERKELEY MOYNIHAN,** C.B., very cordially supported the idea of establishing a local centre of the College, and said he was in profound and confident agreement with everything that Miss Cox-Davies had said. The College was established with the sole object of creating an organisation by which nurses should at last be in a position to govern themselves; that there should be an organised body representative of the interests of nurses; and securing for nurses that collective influence and knowledge and wisdom without which no cause could advance in these days. The object of the College was primarily, he thought, to standardise the efficiency of nursing in this country, and it would do that by securing a certain very brief period—three years was suggested—for the training of nurses in a hospital duly approved and recognised by authority. It would do so, further, by allowing nurses to pass an examination, either through one portal or more, the number being relatively insignificant, and a matter which could be settled around a table within the space of a few minutes. The College would do all these things without infringing upon the tender susceptibilities of other bodies engaged in and dealing with the interests of nurses, the chief object in view being to secure to the public the best that it was possible for the nursing profession to supply.

**Miss COWLIN,** Organising Secretary of the College, urged that nurses joining the College should be surely grounded in the reasons governing their action. In the first place, the College stood for State Registration, and also it was going to form a medium whereby the nursing profession could manage its own affairs and move forward as a self-governing whole, upholding the highest ideals and traditions of the profession. She hoped that unity would be engendered not only through the corporate body, but by the agency of local centres, following the

### COLLEGE OF NURSING: LEEDS MEETING.—(*continued*).

example of this in Leeds. Those who joined the local centre should do so with a sense of great responsibility, with a resolve to attend the meetings regularly, to keep in touch with the nursing press, and to contribute in every way possible to the educational aspect of the centre's work, education being pre-eminently one of the functions of the College. Side by side with all this effort they should never lose sight of the essential spirit of service, without which indeed, higher education would be a vain thing, and improved conditions a luxury. "We want State Registration," she added, "only that we may be able to serve the State more ably and more efficiently."

**The LADY MAYORESS of Leeds** (Mrs. Gott) moved: "That as a result of this meeting a centre of the College of Nursing be formed in Leeds." Speaking for the women of Leeds, she said that any movement designed to improve and regularise the standard of nursing must claim the hearty support of every woman, and she hoped that the proposed centre in Leeds would be a leading light to others in the country.

**Miss LINDALL,** one of the local secretaries of the College, seconded the resolution, saying that already the College had secured 100 members in the district, and she hoped that before long the larger hospitals in Yorkshire would contribute their strength to the movement.

The resolution was then carried amid loud applause.

### GENERAL COMMITTEE AND OFFICERS.

A meeting of those interested in the formation of a local Centre of the College of Nursing was later held at the Leeds General Infirmary. Mr. Charles Lupton was in the chair, and the meeting was addressed by Miss Sparshott, R.R.C., matron of Manchester Royal Infirmary, and a member of the Council of the College, and by Miss Cowlin, Organising Secretary of the College. The following were elected to form a General Committee: Miss Innes, R.R.C., matron Leeds General Infirmary (to be Local Representative for Yorkshire and to stand for General Nursing); Miss Edwards, Maternity Hospital, Leeds (Honorary Treasurer, and to stand for Maternity Hospitals); Miss Lindall, Hospital for Women and Children, Leeds (Honorary Secretary for all hospitals and associations, and to stand for Special and Children's Hospitals); Miss Spann, Township Infirmary, Leeds (Honorary Secretary for Poor-Law and Fever Hospitals, and to stand for Poor-Law); Miss Brothers, 22 Clarendon Road, Leeds (Private Nurses); Miss Barnes, A.R.R.C., District Nurses' Home, Lovell Street, Leeds (District Nursing); Miss Tomlin, R.R.C., East Leeds War Hospital, Leeds (Fever Nurses); Miss Webster (School Nurses); Miss Brandreth (Public Health); Miss Pressland, Clayton Hospital, Wakefield (General Nursing); Miss Binns, Royal Infirmary, Hull (General Nursing); Miss Barry, R.R.C., Huddersfield Royal Infirmary (General Nursing); Miss M. K. Steele, York County Hospital (General Nursing); The Matron, Halifax Royal Infirmary (General Nursing); Miss Foggett, Bradford War Hospital (General Nursing); Superintendent Nurse, North Bierley Workhouse Infirmary, near Bradford (Poor-Law Nursing); Miss Goodrich, North Riding District Nurses' Home, Northallerton (District Nurses and others).

Out of this General Committee a small Executive Committee is to be chosen. It was decided that the Centre should be self-supporting as far as possible, and that members should be asked to pay an annual subscription of 2s. 6d. A course of post-graduate lectures is under consideration.

